# A UK survey of nutritional care pathways for patients with COVID‐19 prior to and post‐hospital stay

**DOI:** 10.1111/jhn.12896

**Published:** 2021-05-12

**Authors:** Victoria Lawrence, Mary Hickson, C. Elizabeth Weekes, Anna Julian, Gary Frost, Jane Murphy

**Affiliations:** ^1^ Faculty of Health and Social Sciences Bournemouth University Bournemouth UK; ^2^ Plymouth Institute of Health Research University of Plymouth Plymouth UK; ^3^ Nutrition & Dietetics Guy’s & St Thomas’ NHS Foundation Trust London UK; ^4^ University College London Hospitals NHS Foundation Trust London UK; ^5^ Nutrition and Dietetics NHS Glasgow and Clyde Glasgow Royal Infirmary Glasgow UK; ^6^ Nutrition and Dietetic Research Group Imperial College London London UK

**Keywords:** COVID‐19 infection, nutrition, care pathway, dietitians, nutritional care

## Abstract

**Background:**

During the global COVID‐19 pandemic, UK dietitians have delivered the best care to help patients recover from the infection. The present study examined the development and evaluation of care pathways to manage nutritional care of patients following COVID‐19 infection prior to and after discharge.

**Methods:**

Registered UK dietitians completed an online questionnaire comprising 26 questions about the development of a pathway, its use, evaluation and training needs.

**Results:**

Of 57 responses from organisations, 37 (65%) were involved in the planning/management of nutritional care. Only 19 responses had a new or adapted COVID‐19 pathway. Of these, 74% reported involvement of dietetic services, 47% reported > 1 eligibility criteria for pathway inclusion and 53% accepted all positive or suspected cases. All respondents used nutritional screening, first‐line dietary advice (food first) and referral for further advice and monitoring. Weight and food intake were the most used outcome measure. All pathways addressed symptoms related to nutrition, with the most common being weight loss with poor appetite, not being hungry and skipping meals in 84% of pathways. Over half of respondents (54%) planned to evaluate their pathway and 83% reported that they were ‘very or reasonably confident’ in their team's nutritional management of COVID‐19. Less than half (42%) reported on training needs.

**Conclusions:**

Despite challenges encountered, pathways were developed and implemented. Dietitians had adapted to new ways of working to manage nutritional care in patients prior to and after discharge from hospital following COVID‐19 infection. Further work is needed to develop strategies for evaluation of their impact.

## Graphical abstract

The present study examined the development of care pathways by UK dietitians to manage the post‐hospital nutritional care of patients following COVID‐19 infection and the evaluation of these pathways. Of the responses, 51% reported developing or adapting a pathway for COVID‐19 infection and 54% planned to undertake evaluation of their pathway. Despite challenges encountered, dietitians have responded rapidly and adapted to new ways of working.

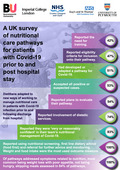

## INTRODUCTION

Nutrition is a crucial part of the recovery process for all patients with COVID‐19, particularly for those who have experienced cardiac or pulmonary complications, as well as for cases where frailty, sarcopenia and malnutrition have developed or been exacerbated.^1^ By August 2020, more than 95 000 patients with COVID‐19 infection had been cared for in hospitals across England alone^1^ and, although the majority of patients may have recovered from the acute phase and been discharged from hospital, the focus has turned towards their recovery as the longer‐term effects of the virus and its treatment become evident.

COVID‐19 infection presents with a diverse range of symptoms that may adversely impact on nutritional status in patients. These include changes in taste and smell, loss of appetite and gastrointestinal symptoms such as diarrhoea and vomiting.^2^ This poses new challenges for the nutritional care of patients who have experienced COVID‐19 infection. National Health Service (NHS) England recognises the role of the dietitian in ensuring adequate nutrition and hydration to prevent malnutrition in patients following hospital discharge in their report ‘After‐Care Needs of Inpatients Recovering from COVID‐19’. In May 2020, the British Dietetic Association (BDA) published the ‘Nutrition and the COVID‐19 Discharge Pathway’^3^ emphasising the importance of screening for malnutrition in patients with COVID‐19 infection. It also called for policy makers, as well as healthcare and dietetic leaders, to take action to ensure that patients have access to appropriate nutrition, with expert guidance from dietitians as part of multidisciplinary rehabilitation pathways. The European Society for Parenteral and Enteral Nutrition (ESPEN) has also produced clinical guidance to inform healthcare rehabilitation pathways to ensure that nutrition is considered at every stage of the patient's journey.^4^ As information about COVID‐19 infection accumulates, there remains a need to develop the evidence to inform new rehabilitation pathways and thus optimise recovery and reduce the likelihood of further deterioration. Care pathways have been used in the NHS from the mid to late 1990 s onwards and are regarded mechanisms for ensuring patient safety, equity in the quality of treatment, optimal use of resources, and a way to improve the efficiency and effectiveness of the care process by integration. They are designed to be a helpful tool for routing patients through the system and are regarded as patient‐centred by allowing individualisation. They plot out the optimal course of treatment for an illness with prompts for relevant interventions by different professionals, and as such are ideally multidisciplinary.^5^ Flexibility and adaptability are paramount.^6^ Therefore, as an initial step towards identifying best practice to inform new care pathways, we report the findings from a national survey. The survey aimed to provide new information about nutritional care pathways to help manage patients with COVID‐19 prior to and following discharge from hospitals. The key research questions were:

- What nutritional care pathways have been implemented by dietitians or their organisations to manage patients with COVID‐19 infection prior to and post‐discharge?
- Have these pathways of care been adapted from other established pathways, or are new pathways being developed?
- Which patients are being targeted by the pathway and why?
- Which elements are included in the pathway of care and how are they measured?
- Are pathways being evaluated and how?
- What are dietitians’ views on the efficacy of the pathways and how confident are they in managing the nutritional consequences of COVID‐19 infection?

## METHODS

The present study employed a cross‐sectional, anonymous online survey of UK dietitians. Ethical approval was obtained from Bournemouth University's Research Ethics Committee (ID 32676).

### Questionnaire development

A questionnaire consisting of 26 questions was developed for the study by the project team. The questionnaire was divided into six main sections: (i) eligibility and respondent details; (ii) pathways related to the nutritional management of patients with COVID‐19 infection; (iii) assessment of nutritional status and specific symptoms that could influence nutritional status; (iv) advice provided; (v) outcome measures used; and (vi) plans for evaluation and training needs. The survey questions included a combination of open and closed questions with categorical responses and Likert scales to rank responses about perception of using the pathway and confidence in the nutritional management of patients with COVID‐19 (see Supporting information, Doc. S1).

Face and content validity were established by piloting the questionnaire with subject experts (*n* = 6) and clinical dietitians (*n* = 6). Subject experts assessed the content validity of the questionnaire and nominated clinically practicing dietitians to assess face validity to ensure clarity, readability and comprehension, as well as time taken to complete the questionnaire. All dietitians were based in England and worked in a combination of settings, including three from community, two from hospital, and one from hospital and community settings. Three of the six dietitians specialised in the care of older adults, two in community services and one in respiratory medicine. Amendments to the survey highlighted during the piloting phase were made prior to national distribution. The survey could be completed within approximately 15–20 min. The online survey JISC Online Surveys©^7^ was used for distribution.

### Sampling and recruitment

A convenience sample of UK Health and Care Professions Council registered dietitians and active members of the BDA formed the sampling frame. Inclusion criteria were dietitians involved in the planning and/or management of the nutritional care of patients with COVID‐19 infection at their Trust or Health Board. Exclusion criteria comprised non‐practising dietitians, retired dietitians, paediatric dietitians, exclusively academic dietitians, student dietitians and dietitians practising outside of the UK. Dietitians were invited to complete the survey via an e‐mail by the BDA and a survey link shared via social media platforms and direct email to BDA Special Interest Groups. Reminders were shared via social media platforms three times per week during the time that the survey remained open. Only one response per organisation was required and therefore participants were asked to complete the survey and to discuss with colleagues on behalf of their organisation. A PDF version of the survey was made available to download so that a collaborative response could be achieved per pathway by an organisation. Information for potential participants was provided on the front page of the survey and respondents were asked to acknowledge they had read this information before completing the survey. Consent was presumed through participation in the survey and all responses were anonymous. The survey was open between 22 June 2020 and 12 July 2020, approximately 3 months after the COVID‐19 outbreak in the UK.

### Statistical analysis

Descriptive statistics are reported such as frequencies for the categorical data using Excel for Office 365 (Microsoft Corp.). Free text responses were listed verbatim then categorised by the research team using qualitative content analysis.^8^

## RESULTS

**FIGURE 1:**
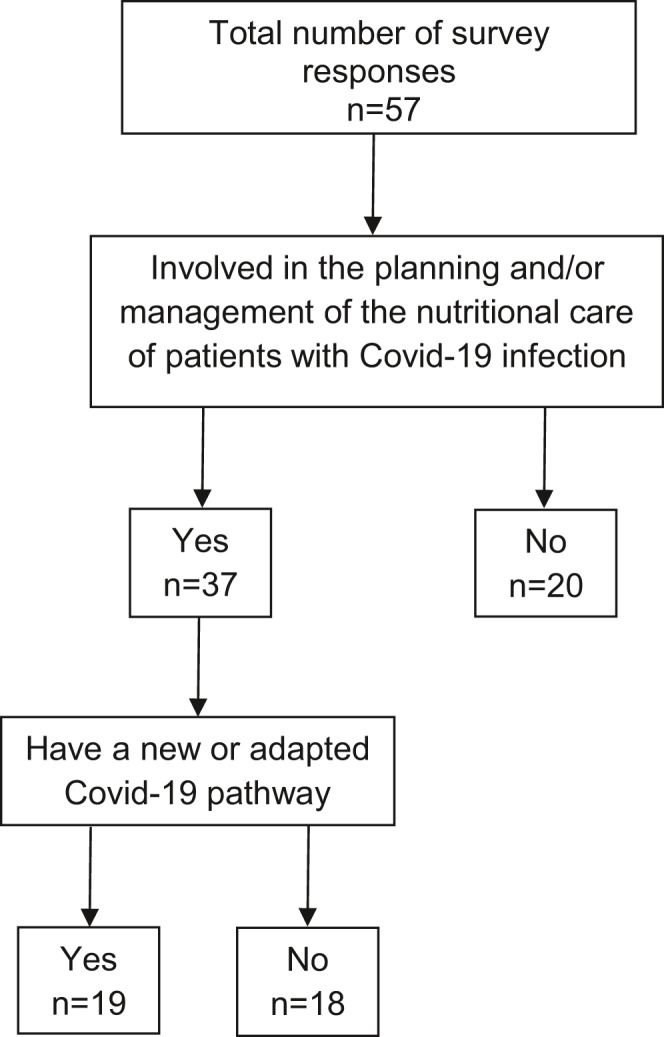
Number of respondents involved in planning and/or management of nutritional care and have a COVID‐19 pathway for patients with COVID‐19 infection

In total, 57 responses were received. Of these, 37 (65%) respondents were involved in the planning and/or nutritional management of patients with COVID‐19 infection (Figure 1). There were 19 respondents who had a new or adapted COVID‐19 pathway for the nutritional care of patients with COVID‐19 infection. The main characteristics are shown in Table 1.

**TABLE 1:** Summary showing the characteristics of the survey respondents involved in the nutritional management of patients with COVID‐19 infection (*n* = 37)

	*n*	%
Country		
England	27	73
Scotland	7	19
Wales	2	5
Northern Ireland	1	3
Clinical setting		
Hospital	13	35
Community	11	30
Hospital and community	11	30
Mental health	1	2.5
Medicines optimisation	1	2.5
Speciality		
Community care	13	35
Critical care	5	14
Other^a^	5	14
Non‐clinical management	4	11
Care of older adults	4	11
General medicine	3	8
Multiple specialities	3	8

^a^ Paediatrics, oncology, mental health, rehabilitation, catering.

### Pathways related to patients with COVID‐19

The different approaches to developing a pathway of care for patients recovering from COVID‐19 infection are shown in Table 2. Eleven (30%) of the 37 respondents reported developing a new or adapting an existing dietetic pathway and eight (22%) had developed a new or adapted an existing multidisciplinary pathway. Eight (22%) of 37 respondents were in the process of developing a pathway, six (16%) had made no changes and four (11%) wanted to implement a pathway.

**TABLE 2:** Pathway approaches reported and professional involvement in developing or adapting a pathway (*n* = 19 respondents)

Yes – it is a new dietetic‐specific pathway developed specifically for COVID−19 patients	Yes – it is an adapted dietetic‐specific pathway of care (e.g., frailty, pulmonary, general rehabilitation, etc.)	Yes – it is a new MDT pathway developed specifically for COVID−19 patients	Yes – it is an adapted MDT pathway of care (e.g., frailty, pulmonary, general rehabilitation, etc.)
Dietetics department	Advanced specialist dietitians from ITU and medical teams	Dietetics, physical health lead nurse	Clinical service lead therapist surgery, trauma and orthopaedics, cancer, respiratory
Community and acute clinical leads	Nutrition and dietetics	Adult dietetics	Adapted post critical care rehabilitation pathway
Dietetic clinical leads for acute, nutritional support and mental health		Physiotherapists, occupational therapists, dietitians, speech and language therapists and social services – new respiratory rehabilitation pathway	Physiotherapists, speech and language therapists and dietitians – under development
Hospital dietitians Prescribing support dietitian CCG		Respiratory rehabilitation pathway	
Hospital nutrition and dietetic services Community nutrition and dietetic services		New AHP Integrated rehabilitation pathway – dietetics, psychology, physiotherapists, speech and language therapists, occupational therapists and podiatry	
Adapted dietetic pathway			
Dietitian and input for MDT pathway for post COVID patients			
Acute dietetic services			
Clinical dietetic leads in acute and community settings			
9	2	5	3

Abbreviations

CCG, clinical commissioning group; ITU, intensive therapy unit; MDT, multidisciplinary team.

Of the 19 participants who had developed a new or adapted a pathway, 10 (53%) respondents included patients who were COVID‐19 positive or who had suspected infection and nine respondents (47%) reported a range of more than one eligibility criteria for inclusion onto their pathway (Table 3).

**TABLE 3:** Criteria for inclusion in the COVID‐19 pathways of care from nine respondents

Inclusion criteria^a^	
‘MUST’ score of 2 or more	3
Subjective methods (deemed to be at risk of malnutrition e.g., poor oral intake or reduced appetite)	3
Enteral tube feeding	2
Patient consistently scoring 1 or above on MUST (minimum one month between screening or bi‐weekly as appropriate)	1
Length of stay in ICU of 4 or more days	1
Patients with COVID−19 infection seen by dietitian prioritising those in ICU and those who required oxygen on ward	1
Dysphagia and/or strictures requiring texture modification, assessed by a Speech and Language Therapist	1
On an Oral Nutrition Supplement prescription (regardless of weight/ weight loss)	1
BMI < 18.5 kg m^–2^	1
BMI < 205 kg m^–2^ with unintentional weight loss of > 5% over the past 3–6 months	1
Patient of any BMI who presents with ≥ 10% unintended weight loss over the previous 3–6 months	1
All admitted in‐patients with COVID−19	1

Abbreviations

BMI, body mass index; ICU, intensive care unit; MUST, Malnutrition Universal Screening Tool.

^a^ More than one criteria was reported.

**FIGURE 2:**
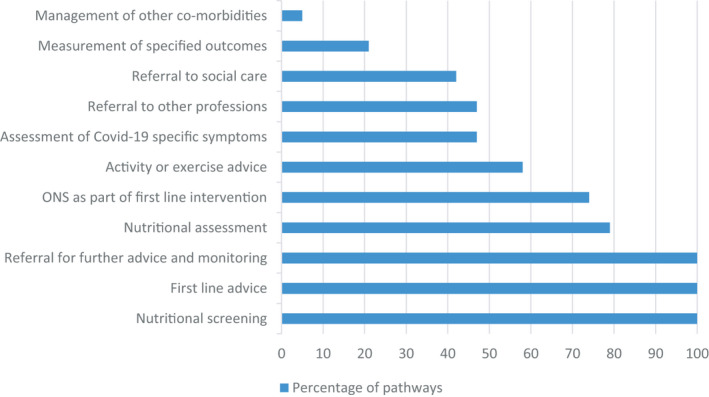
Aspects of nutritional care included in new or adapted nutritional care pathways for patients following COVID‐19 infection (*n* = 19). ONS, oral nutritional supplement

The content of pathways varied, although all pathways included nutritional screening, first‐line nutrition advice and referral for further nutrition advice and monitoring (Figure 2). Most pathways included nutritional assessment and oral nutritional supplements (ONS) as part of first‐line intervention, activity or exercise advice. Less than half assessed COVID‐19 infection‐specific symptoms, or patients patients referred to other professionals or to social care.

### Nutritional screening and assessment

Table 4 shows the nutritional screening and assessment tools reported in the pathways. The majority used the Malnutrition Universal Screening Tool (‘MUST’)^9^ and almost all used the ABCDE (Anthropometric, Biochemical, Clinical, Dietary, Environmental) process for nutrition assessment.

**TABLE 4:** Nutritional screening and assessment tools used for the nutritional care pathways for patients with COVID‐19 infection (*n* = 19)

	*n*	%
Nutritional screening		
‘MUST’	14	73
Patients Association Nutrition Checklist	2	11
Combination of ‘MUST’ and local tool	1	5
Local tool	1	5
WASSP^a^	1	5
Nutritional assessment		
ABCDE^b^	17	90
Patients Association Nutrition Checklist	1	5
Electronic patient records	1	5

Abbreviation

MUST, Malnutrition Universal Screening Tool.

^a^ Weight, Appetite, Ability to eat, Stress factors, Pressure sores/wounds.

^b^ Anthropometric, biochemical, clinical, dietary, environmental.

### First‐line advice

All respondents reported using written or online food first information and the use of locally developed resources. Other resources reported were those available from the BDA, Nutrition and Diet Resources UK , Malnutrition Pathway COVID‐19 and Malnutrition Task Force/Age UK (https://www.malnutritiontaskforce.org.uk/coronavirus‐information‐hub). A variety of ONS were prescribed (see Supporting information, Doc. S2).

### Monitoring of specified outcomes

All 19 respondents reported on the outcome measures monitored routinely in the pathway. The outcome measures monitored depended largely on the setting (e.g., critical care, general ward or community). Weight was monitored as an outcome in 17 (89%) pathways and food intake was monitored in 14 (74%) pathways. Of these, nine (64%) respondents used diet charts or tables and seven (50%) used dietary recall. Patient‐specified goals were measured in 50% of all pathways. Activities of daily living were monitored in six (33%) pathways, physical function in five (28%) pathways and handgrip strength in two (11%) pathways. Two (11%) respondents noted the difficulty in recording outcome measures as a result of virtual clinics. Three respondents reported using mid upper arm circumference (MUAC). However, other outcomes commonly undertaken by dietitians including MUAC were measured by other healthcare professionals such as nursing staff because of restricted access to the wards for dietitians.

### Assessment of COVID‐19‐specific symptoms

**FIGURE 3:**
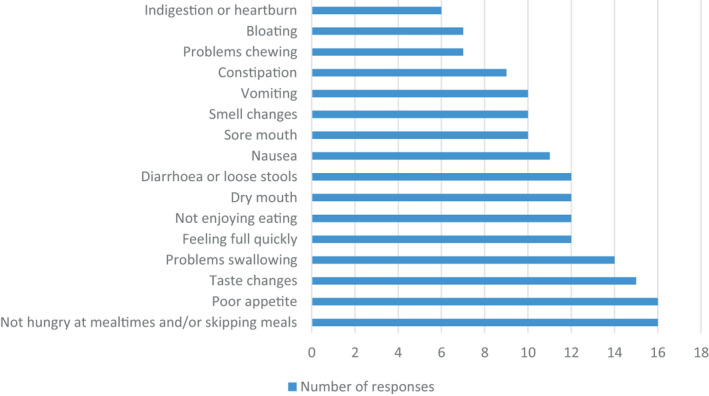
Assessment of nutrition‐related symptoms included in nutritional care pathways for patients following COVID‐19 infection (*n* = 19)

Nineteen respondents reported on the assessment of COVID‐19 symptoms related to nutrition (Figure 3). A variety of symptoms were assessed in the majority of pathways, including not hungry at mealtimes and/or skipping meals (84%), poor appetite (84%) and taste changes (79%). Symptoms less likely to be assessed were indigestion or heartburn (32%), bloating (37%) and chewing problems (37%).

**FIGURE 4:**
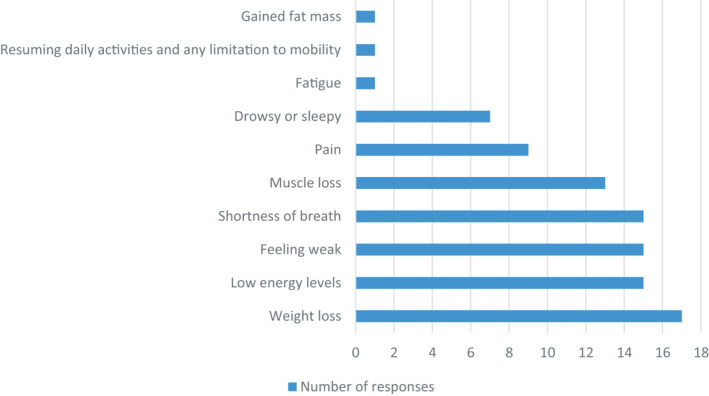
Assessment of physical or functional symptoms associated with COVID‐19 infection (*n* = 19)

**FIGURE 5:**
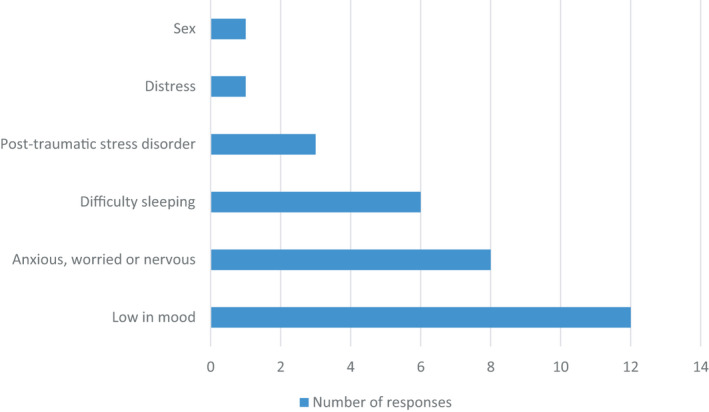
Assessment of emotional or psychological related symptoms associated with COVID‐19 infection (*n* = 19)

Figure 4 shows that the most frequently assessed physical or functional symptoms were weight loss (90%), energy levels (74%), weakness (74%), shortness of breath (74%) and muscle loss (68%). Other symptoms such as pain and feeling drowsy or sleepy or fatigued were less likely to be assessed as part of the pathway. The most frequently assessed emotional or psychological symptoms were low mood (63%), anxiety (42%) or sleep disorders (32%) (Figure 5).

Regarding the provision of advice or resources with respect to management of COVID‐19‐specific symptoms, 12 (63%) respondents reported this for eating and drinking with breathlessness, 10 (53%) respondents reported this for managing loss of taste and smell, 10 (53%) respondents reported this for managing a dry mouth, 10 (53%) respondents reported this for prescription of ONS, seven (37%) respondents reported this for advising on purchasing nutritional supplement drinks, six (31%) respondents reported this for managing diarrhoea or other gastrointestinal disturbances and one (5%) respondent reported this for recommending multivitamin and mineral supplements.

### Evaluation of the pathway

Ten (54%) of the respondents planned to evaluate the pathway. Approaches reported for evaluating the pathways comprised monitoring and review via colleagues about the discharge and review process; patient satisfaction in clinics; staff feedback based on qualitative and quantitative feedback; patient reported outcomes and retrospective audit of pathways based on key performance indicators, such as number of referrals, patients reviewed in virtual clinics, patient symptoms, nutrition support interventions used and assessment of patient perceptions of virtual clinics.

### Confidence in using the pathway

Eight (42%) respondents reported that the pathway was working ‘reasonably well’ and nine (47%) preferred not to comment on the effectiveness of the pathway as a result of the short length of time that it had been in place. Two (11%) respondents, who had created a new multidisciplinary pathway, reported that these pathways were not working well. The first was based in critical care and a lack of resources for the large number of patients was suggested as the main reason why the pathway was not working well. The second pathway was involved in the management of community services, and issues surrounding remote working, such as educating staff and raise awareness of the pathway, were considered to be barriers to the success of their pathway.

### Main difficulties setting up or adapting pathway

Of the 19 respondents who developed or adapted a pathway, 18 reported the difficulties they faced setting up or adapting the pathway (Table 5). These difficulties included issues related to remote working, work pressures, redeployment of dietitians to other roles not related to nutritional care and reduced access to wards for dietitians (possibly as a result of limits on personal protective equipment).

**TABLE 5:** Main difficulties associated with setting up or adapting a nutritional care pathway for patients with COVID‐19 infection from 18 respondents

Difficulty^a^	*n*	%
Remote working including setting up virtual clinics	4	22
High volume of patients and lack of staff/redeployment of staff	3	17
Time constraints for planning and training staff	3	17
Working with other specialities to standardise pathways and provide support and clarification on what advice can be provided	3	17
Long‐term planning – funding and management	3	17
Missed referrals	2	11
Staff adherence to the pathway	2	11
Agreeing outcome measures	1	6
Other issues not related to COVID−19	1	6

^a^ More than one difficulty was reported.

Remote working issues included difficulties communicating with other teams to decide outcome measures, IT difficulties in setting up virtual clinics and the inability to see patients face‐to‐face. Work pressures were linked to low staff and high patient numbers, resulting in other priorities (such as medical emergencies) taking precedence over adherence to the pathway. The redeployment of staff and reduced access to acute wards for dietitians meant that there was less ability to see patients face‐to‐face, there were less dietitians to meet the need for nutritional assessment and providing training, and completing referrals was time consuming. A further challenge faced was creating a pathway that could be standardised across all specialities and multidisciplinary teams. Respondents indicated that their role and responsibilities was unclear and more clarification was needed on the advice and actions taken by all members of the multidisciplinary team involved within the pathway.

Six respondents reported how they overcame difficulties. This included reiterating the existing systems to lead nurses, including updates (written, verbal, online) on nutrition, food and fluid guidance, and offering telephone support to the wards. One respondent indicated that they ‘streamlined’ patients into appropriate clinics for timely nutrition support and another reported prioritising patients and working more closely with physiotherapy.

### Confidence in the nutritional management of patients with COVID‐19 infection

All 37 respondents answered the question about confidence. Seven (19%) reported that they were ‘very confident’ in their nutritional management of patients with COVID‐19, 24 (65%) were ‘reasonably confident’ and six (6%) were ‘neutral’.

### Training needs

Twenty‐four respondents reported on training needs. Five (21%) wanted further training on referral processing to and within community settings to ensure patients receive required support, especially with remote delivery of pathways. Five (21%) wanted training on the long‐term complications of COVID‐19 and how best to support complex patients, such as those with dysphagia or gastrointestinal cancers.

Three (13%) respondents reported that continued research is required and two (8%) reported that mandatory training and the upskilling of non‐acute staff to an intensive care unit settings was required. Five (21%) respondents indicated that training should take a multidisciplinary approach and that nutrition training and awareness should be available to non‐nutrition staff.

Six (25%) respondents reported that they had found the BAPEN and BDA resources useful and that both organisations could be approached regarding the potential use of their resources as the basis of future training programmes. Six (25%) respondents suggested the type of training that they would like included online resources and webinars to support their learning needs.

## DISCUSSION

The present study is the first of its kind to examine nutritional care pathways for patients prior to and following discharge from UK hospitals following COVID‐19 infection. We have provided new information on what care pathways (multidisciplinary and dietetic specific) had been implemented or were planned for development indicating the urgent need for new pathways or the adaption of current pathways. The major findings were significant inconsistencies in the development and content of the pathways and, at this stage, the evaluation of impact of the pathways did not appear to be prioritised or planned. Neither of these findings are surprising given the nature of the global pandemic and the need to respond rapidly to an urgent and critical situation. Nevertheless, COVID‐19 infection will remain prevalent in the community and so it is worth revisiting the pathways initiated speedily to review, evaluate and modify them with respect to incorporating acquired knowledge and experience.

The survey showed no consistent approach to pathway development; there were both dietetic‐specific and multiprofessional pathways developed. Most were newly developed rather than compriising the adaptation of current pathways, which might suggest that the extreme situation could not be successfully mapped onto other existing pathways, or that other pathways simply did not exist. Nevertheless, a minority of respondents did adapt existing pathways, such that, in some areas, this was clearly a possibility or seen as the most efficient approach. Because a care pathway for COVID‐19 infection should map the patient's journey to recovery, it is inevitable that it will cross care settings and involve a variety of professional groups. Thus, it might be more effective to take a multidisciplinary approach, although the critical situation that the pandemic presented may have resulted in a more pragmatic approach, with a care pathway for a single discipline being easier to develop than one involving many professions. Further work needs to explore the contribution of dietitians and the different professions involved in the development or adaptation of pathways and their responsibilities.

The survey attempted to examine the elements of the pathways in some detail. There was a lack of consistency in the criteria for inclusion of a patient on the pathways, where criteria beyond a positive or suspected COVID‐19 infection were used. Criteria included both subjective (poor appetite or intake, etc.) and objective measures (‘MUST’ score, low body mass index, intensive care unit admission for a specified period). Criteria are useful to target treatments to patients most in need and, in these care pathways, the prevention of malnutrition was the key outcome. Thus, criteria to select those most at risk of subsequently developing malnutrition were used. Because the complications and outcomes of COVID‐19 infection were largely unknown at the time that these pathways were developed, many respondents had decided that all infected patients should be seen regardless of other risk factors. However, differences observed in the criteria for inclusion of patients onto the pathways indicates a lack of clarity about which patients will benefit most. Therefore, there is a need for more research aiming to understand the consequences of COVID‐19 infection better and to standardise the approaches used nationally.

Consistent features of all of the pathways were nutrition screening, use of first‐line dietary advice, and referral for further nutrition support and monitoring. Because these are important and basic steps in the assessment and treatment of people at risk of malnutrition,^10^ it is unsurprising these were used in all pathways. Furthermore, evidence suggests that nutritional support can prevent or help to reverse the problems associated with undernutrition.^11^ The most commonly used screening tool was ‘MUST’ and, because this is the most widely used tool in the UK,^12^ this is to be expected. In addition, screening and nutritional support are the first two statements in the ESPEN guidance for nutritional management of COVID‐19 infection, a practical document published early in the pandemic.^4^

Similarly, more detailed nutritional assessment, as well as ONS as part of the first‐line intervention, were used in most pathways. Trials using ONS during acute illness have shown reduced length of hospital stay^13^ and reduced re‐admission.^14^ Additionally, in respiratory disease (chronic obstructive pulmonary disease ), ONS have been shown to improve peripheral muscle strength^15^; thus, there is good evidence for this element in these pathways. However, compliance with ONS prescriptions is variable^16^ and so on‐going dietetic support may be needed to optimise consumption.

Interestingly, more than half the nutritional care pathways specified including advice on activity or exercise. Muscle mass is crucial for health and independence^17^ and it is lost during ageing.^18^ The best evidence for retention of muscle mass and regain after loss is resistance exercise.^19^ Thus, there is good evidence that exercise should be a part of optimal recovery for patients who have had reduced mobility, as well as reduced nutritional intake.

Less than half the pathways specifically assessed COVID‐19‐related symptoms, referred to other professions or social care, measured specified outcomes, or attempted to manage other co‐morbidities. This may reflect the novel and urgent nature of the situation resulting in a need for a rapid development of pathways and a lack of specific information about COVID‐19 symptoms. However, given reports about the negative impact of COVID‐19 on food insecurity,^20^ it is important that integrated care pathways with social care are developed as part of post‐discharge nutritional care. Taste changes, one of the key symptoms of COVID‐19 infection,^21^ was specifically included in most pathways.

The most commonly monitored outcomes in the pathways were body weight and food intake. These are important and obvious indicators of the response to nutritional treatment, or further deterioration in condition and a rising risk of malnutrition. We did not investigate how body weight was measured or self‐reported. However, the data indicate that other tools were used to identify risk of malnutrition, such as the Patients Association Nutrition Checklist, which does not require a measure of body weight. Although a recent study shows the potential for e‐scales in clinical practice,^22^ further studies need to explore the validity of collecting measures of body weight virtually or the use of a validated screening tool that does not include measures of body weight. Far fewer pathways measured functional measures, such as hand‐grip strength and activities of daily living, and others took the approach of using achievement of patient specified goals. There appeared to be considerable problems in obtaining outcome measures, particularly objective measures, as a result of virtual clinics and other infection control measures.

At the time of the survey, 54% of respondents who had developed or adapted a pathway were in the early stages of planning to evaluate their pathways. None reported a plan for evaluating the impact of the pathway on patients. The evaluation of any new service development is important in terms of checking that it is working effectively and achieving the desired goals.^23^ These data indicate that many dietitians find it challenging to design and implement suitable evaluation plans. Some respondents reported that they felt the pathway was working reasonably well, whereas others did not, highlighting the need for on‐going evaluation. The challenges of setting up or adapting the pathways were highlighted and reflect the unprecedented situation that the pandemic generated. Some of the difficulties, such as time constraints and working across disciplines, are not specific to the pandemic but were potentially compounded and intensified in the pressurised working environment that existed. The dietitians involved also demonstrated their problem‐solving capabilities by engaging practical and often simple solutions to the challenges they faced. A frequent drawback of care pathways is that they are less patient‐centred and flexible and often do not embed the patient's voice in their journey through the ‘pathway’.

Most respondents responded that further training about nutrition‐related issues in COVID‐19 infection was required. Most stated specific areas for training, with these being varied, including process issues (referral within the community), other staff training needs (nutrition training for non‐nutrition staff) and their own need for more information (long‐term complications and supporting complex patients). There was a recognition that as new aspects of managing COVID‐19 infection would continue to rapidly evolve, such as long COVID‐19.^24^ New approaches to online training and webinars were received favourably and were considered as an innovative approach for nutrition training that could be further developed.^25^

We recognise the limitations that the survey may not provide a complete picture of practice from Health Trusts and Health Boards in the UK. Although most of the respondents were from England, there was representation from Scotland, Wales and Northern Ireland, as well as from a range of different clinical settings and specialities. The survey was strengthened by respondents having the opportunity to add free‐text answers for most questions. The research was undertaken over a short time frame towards the end of the first wave of the COVID‐19 outbreak (in June 2020), which might have limited the number of dietitians able to respond as a result of time constraints. Nevertheless, although it was too early for some organisations to provide information and some organisations were still in the process or were intending to develop new pathways, these are encouraging developments and require further investigation. Surprisingly, there were 20 respondents who were not involved with the planning and/or management of nutritional care of patients with COVID‐19 infection. Further research is needed to understand whether there has been dietetic involvement in nutritional care for COVID‐19 infection subsequent to the time of the present study, given that there has been a second wave of the pandemic at the end of 2020/early 2021, with greater numbers of people with COVID‐19 infection being admitted to UK hospitals.

## CONCLUSIONS

The present study provides new information on the development of new nutritional care pathways for patients recovering from COVID‐19 infection, as well as what pathways had been implemented to date or were under development. New or adapted post‐hospital discharge pathways will support the transition from hospital to home and particularly benefit those with long COVID‐19. Dietitians have had to respond rapidly and have adapted to new ways of working to overcome the challenges encountered. Further work will use these findings, combined with a review of the current literature, to design an evidence‐based pathway for the management of malnutrition in patients prior to and after discharge from hospital following COVID‐19 infection and to enable more consistency and standardisation of practice.

## CONFLICT OF INTERESTS

The authors have no conflicts of interest.

## AUTHOR CONTRIBUTIONS

All authors contributed to the conception, design and piloting of the questionnaire and interpretation of the data. VL designed, implemented and analysed the online questionnaire. VL, JM and MH drafted the manuscript. All authors critically reviewed the content of all drafts and have approved the final version of the manuscript submitted for publication.

## ETHICAL APPROVAL STATEMENT

Ethical approval was obtained from Bournemouth University’s Research Ethics Committee (ID 32676).

## TRANSPARENCY DECLARATION

The lead author affirms that this manuscript is an honest, accurate and transparent account of the study being reported and that no important aspects of the study have been omitted.

### Peer Review

The peer review history for this article is available at https://publons.com/publon/10.1111/jhn.12896.

## Supporting information

Supplementary Material
