# Supplementary material for: A UK survey of nutritional care pathways for patients with COVID‐19 prior to and post‐hospital stay
Source: J Hum Nutr Diet. 2021 May 12;34(4):660–9. doi: 10.1111/jhn.12896 (PMC8250968; doi:10.1111/jhn.12896)
Supplement: Supplementary file 1 — Supplementary Material [file JHN-34-660-s001.docx]

Supplementary Information 1

| **Survey questions** |
| --- |
| What setting do you work in?  What speciality do you currently work in?  What UK country are you based in?  What County (England, Wales, N. Ireland) or Council Area (Scotland) is your Trust  or Health Board situated in?  Do you have a Covid-19 pathway in your Trust or Health Board?  Please state what pathway you adapted.  Who undertook the development of this pathway?  Do you include criteria for inclusion on the pathway of care?  Please state which patients are eligible for support.  Which of the following elements are included in your pathway of care?  Nutrition screening – what tool do you use?  Nutrition assessment – what tool or framework do you use?  Which possible appetite and gut related symptoms are patients asked about?  Which possible physical or functional symptoms are patients asked about?  Which possible emotional or psychological symptoms are patients asked about?  First line advice – what is the first line advice provided to patients?  Provide details of the resources used.  Provide details of the ONS prescribed.  Please describe what criteria would trigger a referral to the dietitian for further advice and monitoring.  Please describe which other professionals are involved in the pathway and what the criteria are for referral.  Please describe which other services are involved in the pathway and what the criteria are for referral.  What outcome measures are monitored?  Provide details of measurement tools used.  Do you plan to evaluate the pathway?  How do you plan to evaluate it?  What is your current perception of how well the pathway is working?  What have been the main difficulties you have faced in setting up the pathway or  adapting the old pathway, and how have you overcome them?  How confident are you in the nutritional management of Covid-19 patients?  What training, if any, would you like provided to support the treatment of Covid-19 patients? |

Supplementary information 2

| **Food first resources** | **ONS prescription** |
| --- | --- |
| Food first used in community where possible (2) | ONS used in hospital setting and in community where required (2) |
| Food first diet sheets | First line prescribing |
| Advice to ward staff regarding finger foods and energy dense meals | AYMES shake powder |
| Kingston Hospital Covid-19 diet sheet | Promoting first line prescribing of Fodlink complete on discharge form hospital instead of bottled ONS - fortisip compact protein |
| Malnutrition pathway Covid-19 resources | First line formulary |
| Malnutrition pathway post Covid-19 | First line on BNSSG formulary found on remedy online |
| Covid-19 Supporting your Recovery – Developed by Lancashire teaching hospitals (BDA resource) | 1st line is Fortisip compact protein |
| Nutrition at home following critical illness (BDA resource) | Fresubin Energy, Fresuin Jucy, Fresubin 2kcal, Fresubin Protein Energy, Fresubin 3.2kcal |
| Locally developed resource based on BDA guidance resources | Fortisip compact protein is the standard supplement used as part of MUST management plan in hospital  A mixture of supplements depending on patients need |
| Eating well to heal well | Powdered supplements mixed with full fat milk as first line, second line is bottled ready to drink supplements |
| Hydration boosters |  |
| Food first advice |  |
| Eating well after Covid-19 |  |
| NDR-UK Eating Better Feeling Better provided |  |
| Get More In Snacks - NHS Lanarkshrie resource |  |
| NHS Lanarkshire resource - Nutrition advice during and after Covid-19 |  |
| NHS Lanarkshire - Covid -19 Nutrition advice for your recovery |  |
| How to increase your calories - LDH resources |  |
| Food First- How to gain a pound a week |  |
| Malnutrition Covid-19 BDA resources |  |
| Malnutrition Pathway Covid-19 leaflets BDA joint resources |  |
| Information leaflet developed for the health board. Nutrition support information (leaflet and online) already available, insert created to outline the symptoms associated with Covid-19 which may impact on nutrition (as above) and provide information to support eating with symptoms |  |
| Departmental diet sheet entitled 'Eating Well in Hospital'. |  |
| Hospital nutritional support discharge packs (supplement sample, prescription request letter, food first advice information sheet). |  |
| Malnutrition task force |  |
| Locally developed diet sheet: https://keepingmewell.com |  |
